# Improving Mood Through Community Connection and Resources Using an Interactive Digital Platform: Development and Usability Study

**DOI:** 10.2196/25834

**Published:** 2021-02-26

**Authors:** Robin Ortiz, Lauren Southwick, Rachelle Schneider, Elissa V Klinger, Arthur Pelullo, Sharath Chandra Guntuku, Raina M Merchant, Anish K Agarwal

**Affiliations:** 1 Perelman School of Medicine University of Pennsylvania Philadelphia, PA United States; 2 Department of Computer and Information Science, University of Pennsylvania Philadelphia, PA United States; 3 Penn Medicine Center for Digital Health Philadelphia, PA United States

**Keywords:** community, COVID-19, digital health, digital tool, mental health, mood, prospective, thematic analysis, virtual support, well-being

## Abstract

**Background:**

COVID-19 continues to disrupt global health and well-being. In April-May 2020, we generated a digital, remote interactive tool to provide health and well-being resources and foster connectivity among community members through a text messaging platform.

**Objective:**

This study aimed to prospectively investigate the ability of a health system–based digital, remote, interactive tool to provide health and well-being resources to local community participants and to foster connectivity among them during the early phases of COVID-19.

**Methods:**

We performed descriptive and nonparametric longitudinal statistical analyses to describe and compare the participants’ mood ratings over time and thematic analysis of their responses to text messages to further assess mood.

**Results:**

From among 393 individuals seeking care in an urban emergency department in an academic setting, engaged in a two-way text messaging platform, we recorded 287 mood ratings and 368 qualitative responses. We observed no difference in the initial mood rating by week of enrollment [Kruskal-Wallis chi-square *H*(5)=1.34; *P*=.93], and the average mood rating did not change for participants taken together [Friedman chi-square *Q*(3)=0.32; *P*=.96]. However, of participants providing mood ratings at baseline, mood improved significantly among participants who reported a low mood rating at baseline [n=25, 14.97%; *Q*(3)=20.68; *P*<.001] but remained stable among those who reported a high mood rating at baseline [n=142, 85.03%; *Q*(3)=2.84; *P*=.42]. Positive mood elaborations most frequently included words related to sentiments of thankfulness and gratitude, mostly for a sense of connection and communication; in contrast, negative mood elaborations most frequently included words related to anxiety.

**Conclusions:**

Our findings suggest the feasibility of engaging individuals in a digital community with an emergency department facilitation. Specifically, for those who opt to engage in a text messaging platform during COVID-19, it is feasible to assess and respond to mood-related queries with vetted health and well-being resources.

## Introduction

### Background

Since January 2020, COVID-19 has continuously disrupted global health and well-being. The World Health Organization (WHO) declared a state of global public health emergency, which is the highest level of alarm under international legislation. The WHO reported that COVID-19 induces and exacerbates fear, worry, anxiety, loneliness, and stress, especially among vulnerable populations and those with chronic and underlying health conditions [1].

Previous studies have reported that connecting individuals to community resources and providing ongoing support can decrease loneliness, thereby potentially improving well-being in the context of the trauma associated with COVID-19 [2,3]. Additionally, during the pandemic it is especially critical for individuals to be directed to trusted community and health information resources [4]. Given the concerns regarding the mental health impacts of COVID-19, the need for community interventions to address these potential consequences has arisen [5].

Despite the current need to address mental health through community-based interventions, the implementation of such interventions has been difficult owing to public health constraints such as social distancing. This is particularly concerning for vulnerable population (eg, homeless individuals, older individuals, those with substance abuse, or those with mental health issues) who may not have access to traditional support networks or resources because many physical locations are nonoperational, operating through drastically reduced hours, or operating at limit capacity. Digital platforms and technology and their utility have received increasing attention in bridging the gaps between individuals and community-based support [4]. The rapid expansion of digital platforms that offer connection are critical for supporting individuals and their networks to foster access to credible health information and support mental health and well-being during social distancing and shelter-in-place orders [6].

### Objective

This study aimed to prospectively investigate the ability of a health system–based digital, remote, interactive tool to provide health and well-being resources to individuals and facilitate a sense of connection during the early phases of COVID-19. We investigated self-reported individual mood ratings of the study participants and hypothesized that digital engagement and community resources would improve their mood. Additionally, we investigated user-generated comments to identify themes in areas of need.

### Hypotheses

We first hypothesized that texting individuals is feasible and acceptable to disseminate the tool as we continually attempt to define and improve mental health and well-being. Second, given that participants received differential text-based follow-up depending on their initial mood rating with the aim to improve their self-reported mood rating by providing continuous support, we hypothesized that participants beginning the intervention with low self-reported mood ratings would experience mood improvement through the 4-week intervention, while those with initial high self-reported mood ratings would retain such a rating.

## Methods

### Intervention and Participants

In March 2020, we built a digital platform that deployed automated texting to assess well-being and provide the responders with access to community-based health and well-being resources. The platform provided participants with institution-supported health information, COVID-19 updates, virtual support, well-being tips, and additional resources. We used Mosio, a platform complying with the Health Insurance Portability and Accountability Act of 1996, to invite eligible participants and communicate with them through two-way texting. This study was approved by the institutional review board and quality improvement unit of the University of Pennsylvania.

**Figure 1:**
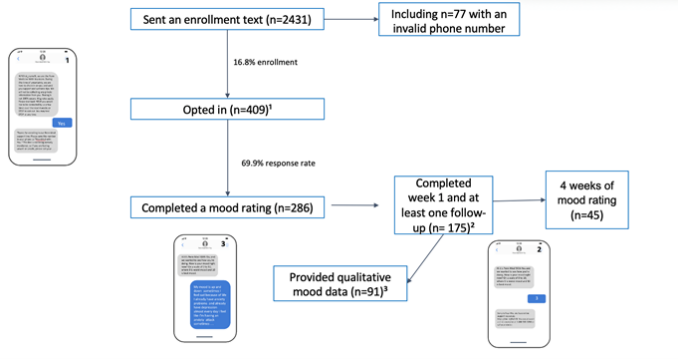
Schematic representation of the study design. A total of 2431 individuals were sent an enrollment text after being discharged from the emergency department at one of two academic medical centers emergency. Of them, 409 (17%) individuals opted in and were asked to self-report well-being, of whom 286 (70%) responded. Of them, 236 (83%) individuals provided a mood rating at week 1 and 175 (60%) responded during week 1 and at least one follow-up, including 101 (35%) individuals who responded at weeks 1 and 4. Furthermore, 45 (16%) participants responded at all 4 weeks.

Eligible participants, including adults aged >18 years and with SMS-capable phone numbers were identified from the electronic health record and approached for enrollment through text message after being discharged from one of two emergency departments (ED) (including a university medical center ED with a high patient capacity, and a second community-based university-affiliated hospital ED with a comparatively lower patient capacity) in the Philadelphia area between April 13 and May 8, 2020. Eligible subjects needed to opt into the follow-up text messages and the 4-week program after enrollment (Figure 1).

Upon enrollment, participants received weekly text messages asking them to rate their mood from 0 to 10 [7]. Individuals could voluntarily provide qualitative descriptors of their mood along with their rating. Tiers of mood rating by the participants trigger the receipt of differential content (Multimedia Appendix 1). Participants reporting a “high” mood rating of 7-10 would always receive a link to an uplifting resource, whereas those reporting a “low” mood rating of 0-3 always received links to mental health and well-being resources. Those reporting a “medium” mood rating of 4-6 received links to either an uplifting resource or to the resource homepage to view all featured resources, alternating each week.

### Statistical Analysis

#### Quantitative Analysis

We used summary statistics to describe the participant demographics. We performed the two-sample Student *t* test and Fisher exact test to compare the demographics of responders and nonresponders. We conducted a quantitative and qualitative analysis of the mood rating data and a descriptive analysis for all mood ratings.

#### Changes in Mood Rating Stratified by Low and High Initial Mood Ratings

We used a Kruskal-Wallis rank test to assess weekly mood ratings of the cohort through the study period (7 weeks) and differences in δ mood (difference in mood rating between weeks 1 and 4) across the three groups of initial mood rating (low, medium, high) and further assessed these differences using the Dunn post hoc test. We performed nonparametric longitudinal statistical analysis using the Friedman test to compare mood ratings by participant over the course of their enrollment in the intervention (4 weeks). To test our hypothesis that participants with an initial “low” mood rating upon initial participation in the intervention would experience mood improvement over the 4-week intervention, we stratified the aforementioned analysis by “low” (rating 0-3) and “high” (rating 7-10) mood.

#### Qualitative Overall Themes

We used a qualitative thematic analysis approach and applied the five stages of framework analysis (familiarization, identification of a thematic framework, indexing, charting, mapping, and interpretation) [8]. We performed thematic coding of the data through an iterative open coding process. An initial set of codes was generated after open review of a subset of responses by the authors (RO and LS), which yielded a codebook (Multimedia Appendix 2). The author (LS) then coded and independently reviewed qualitative responses (n=368) for consistency and agreement.

#### Qualitative Mood Themes

After two authors (RO and LS) applied the initial codes to the main content and reviewed them, we observed that the codes were reproducible and in themselves represented themes from among the main content messages. However, considering our aim to assess mood in this study, we conducted a subanalysis of the content in the text messages specifically under the main theme (code) of “mood” to form subthemes (LS). Word clouds were generated to capture the raw counts of most frequently used words in the open-ended responses for messages identified under the theme of “mood” and subthemes of “positive mood elaborations” and “negative mood elaborations.”

#### Data Exclusion

Over the study period, a total of 2431 individuals were discharged from the ED of one of two urban academic medical centers and approached with an enrollment text. Of them, 409 (17%) opted into the “With You” program, and 383 participants provided demographic (n=374) or mood rating (n=286) data. For completeness, all data available from the 383 samples were used for all statistical analyses.

## Results

### Intervention and Participants

The 409 individuals who opted in the “With You” program were asked to self-report well-being. Almost two-third (n=286, 70%) participants responded (Figure 1). Of them, 236 (83%) responded with a mood rating at week 1, and 175 (60%) responded during week 1 and to the follow-up text during week 4, including 101 (35%) who responded during weeks 1 and 4. Furthermore, 45 (16%) participants responded during all 4 weeks. The majority of participants were Black (n=275, 73.5%), were female (n=264, 70.1%), and had a mean age of 46.9 (SD 16.8) years (Table 1). Most participants (n=225, 60.2%) were recruited from the ED of the hospital with a high overall patient capacity (hospital A). We observed no difference in demographics between mood-rating responders and nonresponders.

**Table 1:** Participant demographics (N=374).

Characteristics		Total population^a^	Responders (n=280)	Nonresponders (n=94)	*P* value	
Age (years), mean (SD)		46.9 (16.8)	47.1 (16.9)	44.1 (17.2)	.06	
Females, n (%)		264 (70.1)	202 (54.0)	62 (16.6)	.26	
**Race, n (%)**						.51
	Black	275 (73.5)	204 (72.9)	71 (75.5)		
	White	69 (18.4)	55 (19.6)	14 (14.9)		
	Asian	14 (3.7)	11 (3.9)	3 (3.2)		
	Other	16 (4.3)	10 (3.6)	6 (6.4)		
Hispanic ethnicity, n (%)		14 (3.7)	10 (3.6)	4 (4.3)	.65	
**Emergency department, n (%)**						.91
	Hospital A	225 (60.2)	168 (60.0)	57 (54.3)		
	Hospital B	149 (39.8)	112 (40.0)	37 (39.4)		

^a^Of the total study sample (n=383), all demographic data were missing for 9 (2.3%) participants.

### Statistical Analysis: Quantitative

**Figure 2:**
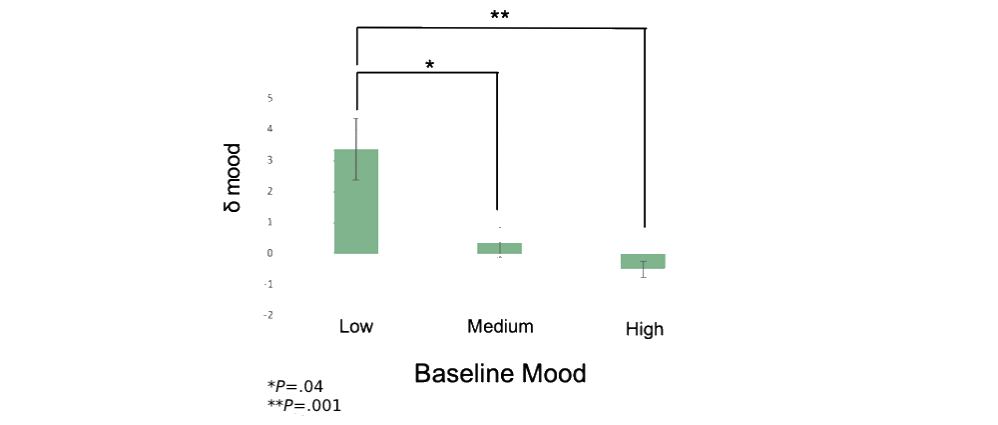
Change in mood from week 1 to week 4 of the intervention (δ) for participants varied significantly across mood tiers [*H*(2)=14.85; *P*<.001]. Pairwise comparisons revealed significant differences between low and medium mood tiers (*P*=.04) and between low and high mood tiers (*P*=.001). *P=.04, **P=.001.

Mood ratings did not significantly change through the course of the study [7 weeks; *H*(5)=1.34; *P*=.93] or by participant when all participant mood ratings were cumulated [*Q*(3)=0.32; *P*=.96]. However, on comparing mood changes between weeks 1 and 4 of enrollment (δ) by grouping participants on the basis of their initial mood rating (low, medium, and high), δ was significantly higher for participants who initially reported a low mood rating [*H*(2)=14.85; *P*<.001] (Figure 2). The Dunn post hoc test revealed significant pairwise differences in δ between low and medium mood tiers (*P*=.004) and between low and high mood tiers (*P*=.001) but not between medium and high mood tiers (*P*=.11). A sensitivity analysis stratified by initial mood, using a dichotomous variable for a low mood rating (0-5, n=69) and a high mood rating (6-10, n=167), did not impact our findings (*z*=2.6; *P*=.009).

#### Changes in Mood Rating Stratified by Low and High Initial Mood Rating

**Figure 3:**
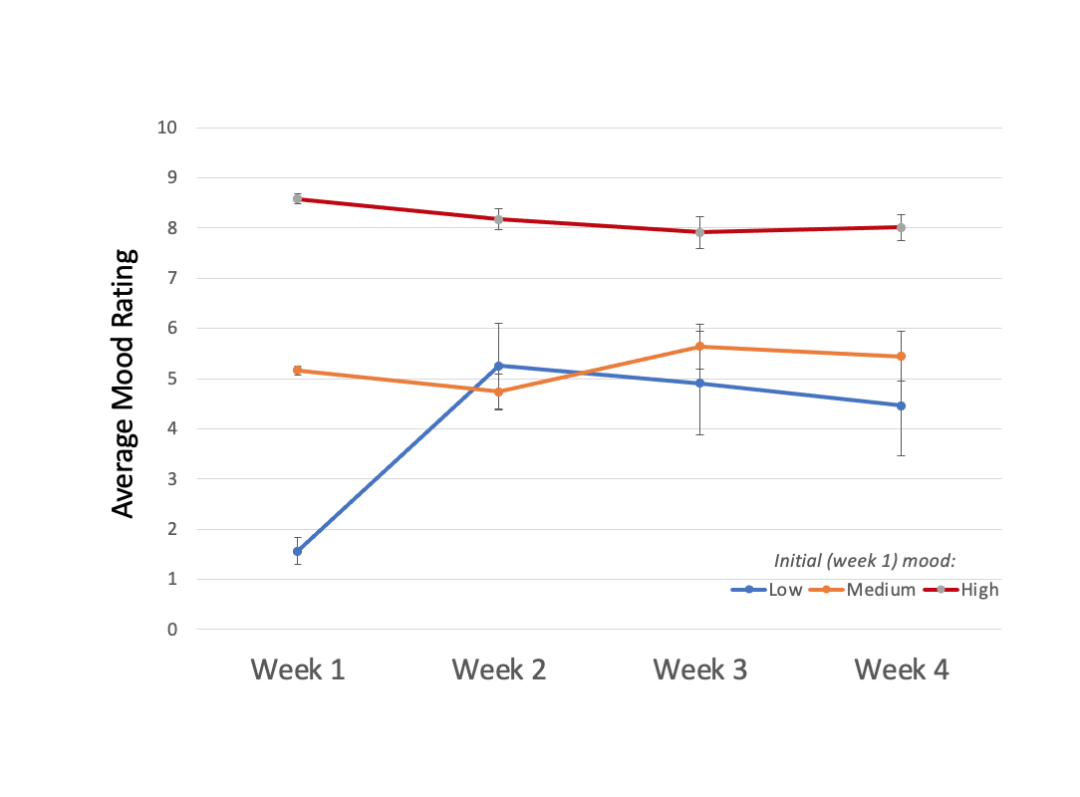
Change in mood from week 1 to week 4 of the intervention (δ) for participants varied significantly across mood tiers [H(2)=14.85; *P*<.001]. Pairwise comparisons revealed significant differences between low and medium mood tiers (*P*=.004) and between low and high mood tiers (*P*=.001).

The remaining quantitative analysis was stratified by a low initial mood rating (0-3) and a high initial mood rating (7-10). Among participants who provided mood ratings at baseline, for those with a low mood rating at baseline (n=25, 14.97%), mood significantly improved through the 4-week intervention [*Q*(3)=20.68; *P*<.001] (Figure 3). Post hoc analysis revealed a significant increase in mood ratings at week 1 and those at weeks 2, 3, and 4 (*P*<.001 for all three pairwise comparisons) but no change in mood rating between weeks 2 and 3 (*P*=.46), 3 and 4 (*P*=.48), and 2 and 4 (*P*=.49). However, among participants who provided mood ratings at baseline, those who reported initial high mood ratings (n=142, 85.03%) retained their high mood rating (showing no longitudinal change) across the 4-week intervention [*Q*(3)=2.84; *P*=.42] (Figure 3).

### Statistical Analysis: Qualitative Overall Themes

Qualitative analysis revealed the main content themes based on 368 qualitative responses to the text messages through the platform (Table 2 and Multimedia Appendix 2), including administrative references, health-related responses or questions, mood elaborations, and brief statements of sentiments or pleasantries. Of these main themes, most (n=135, 36.6%) were related to mood. In total, 125 (34.0%) participants simply reported “pleasantries,” where participants responded with comments such as “have a blessed day” [Participant #1, female, aged 62 years]. Health-related responses were recorded from among 53 (14.4%) responses, including, for example, asking for assistance with health insurance.

**Table 2:** Qualitative elaborations on mood (N=135^a^).

Themes (codes)	Operational definition	Frequency, n (%)	Illustrative examples
Positive mood elaborations	Elaborates on how or why they are in a good or positive mood	71 (52.6)	“I am in a good space. Praying for this attack on America to end so that we can get back to a normal existence. On a scale from 1-10 my mood is a 10. I'm grateful to be alive and I'm trusting God to restore your lives the way that is best for us and pleasing to Him.” [Participant #2, female, aged 58 years] “Should be a 10 later cause of the beautiful weather” [Participant #3, male, aged 39 years] “My mood is much better. I am working with my therapist” [Participant #4, female, aged 43 years]
Somatic	Discusses somatic symptoms	32 (23.7)	“My mood is an 8 right now just came from dialysis and is cramping a little” [Participant #5, male, aged 62 years]
Negative mood elaborations	Elaborates on how or why they are in a bad or negative mood	17 (12.6)	“Thanks still at 3 I am going to call after I talk to my doctor” [Participant #6, male, aged 65 years] “My mood is up and down sometimes I feel sad because of life I already have anxiety problems and already have depression almost every day I feel like I'm having an anxiety attack sometimes …” [Participant #7, female, aged 38 years]
Medical or scheduling concerns	Asks about medical appointments or scheduling related to mental health or mood	5 (3.7)	“I'm going to reach out to my PCP and see if there is anything he can do/let him know what's going on” [Participant #8, female, aged 35 years]

^a^The sample of qualitative responses coded for the overall theme of “mood,” which was used for subanalysis of mood elaborations, including 10 (7.4%) responses that were noncodable through subtheme analysis.

#### Qualitative Mood Themes

Given our interest in understanding mental health, further subanalysis of these 135 responses coded under the main theme of “mood” revealed 7 subthemes among those listed in Table 2. Most subthemes were positive mood elaborations, with a frequently observed association between mood and health-related topics. For example, somatic health concerns included citing pain impacting mood as, “my knees are bothering me this morning but otherwise I am okay” [Participant #2, female, aged 24 years]. Medical health concerns included the need for the participants to reach out to their health care providers (eg, primary care providers or psychiatrists) for medical assistance with their mood:

I really don't know like I said I do have a psych therapist he calls me once a week there is nothing more that can be done for me ... I take my psych meds everyday they help somewhat ...

Participant #3, female, aged 46 years

**Figure 4:**
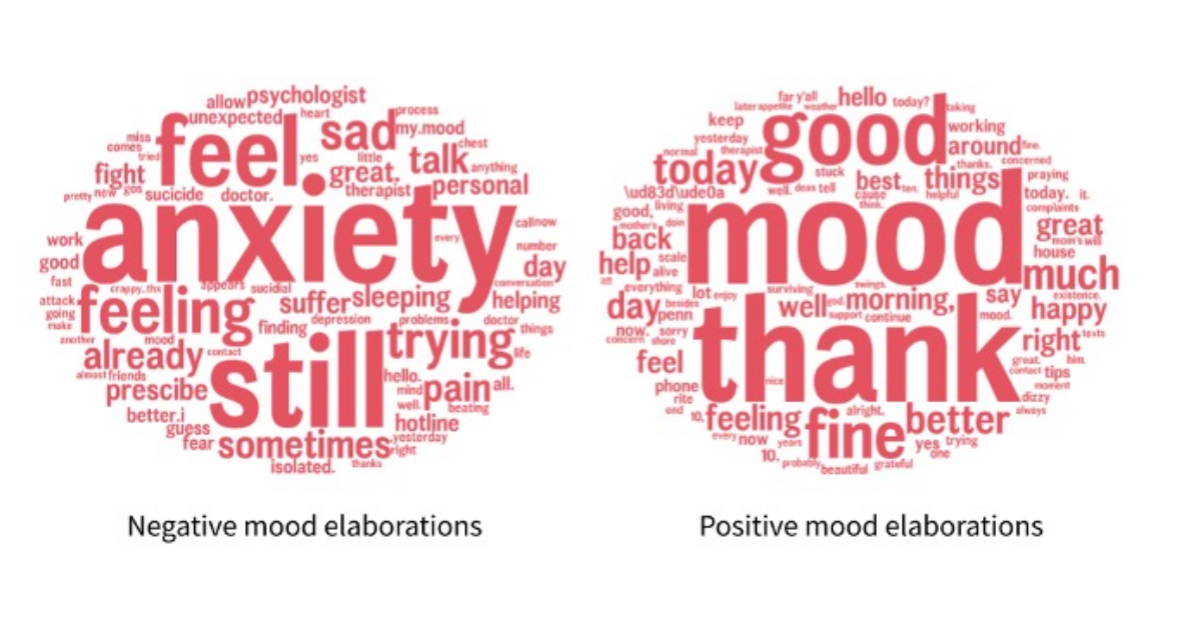
For the respondents who provided qualitative text responses related to mood (n=136), positive mood elaborations were most common (n=71, 52.6%) and frequently referenced sentiments of “good mood” secondary to “thanks.” Negative mood elaborations (n=17, 12.6%) often referencing statements of “anxiety,” “feel,” and “still.”

Analysis of the subthemes for positive and negative mood elaborations provided us a nuanced understanding of how participants reflect and elaborate on their current level of well-being (Figure 4). Among negative mood elaborations, the words “anxiety,” “still,” and “feel” were most commonly used. We did not observe any theme or the use of the word “loneliness,” although 2 participants with negative mood elaborations alluded to isolation from “friends” and “contact,” with one stating, “I miss the personal contact and conversation.” [Participant #11, female, aged 35 years]

Among the positive mood elaborations, the words “thank” and “good” were most commonly used. For instance, several participants sent responses to the text messages, which were similar to the following representative responses:

Thank you for your help tips I also meant to tell you that I'm over 60 years old I also am a diabetic and asthma so it really is taking a toll on me but I do have a in home health care person he helps me out a whole lot but when I am by myself that's when I feel the worst so I will keep up with the tips and keep up with your team thank you.

Participant #4, female, aged 63 years

I [am] feeling a lot better thank you so much for being here for me.

Participant #5, female, aged 57 years

Table 2 provides additional examples of mood elaborations. 

## Discussion

### Principal Findings

Our study demonstrates the feasibility of using a novel digital texting platform to assess mood early during COVID-19 and the ability to acutely identify and address trends specifically supporting mental health and well-being among individuals recruited from the EDs of two hospitals. First, we demonstrate the feasibility of enrolling individuals to the digital platform in an otherwise less accessible population for consistent communication with the health system. The ED is a unique recruitment site with varying medical conditions, chief complaints, and a patient population often without access to or engagement in consistent preventive care. Nonetheless, individuals discharged from the ED, who opted into this study, were highly engaged. Our overall response rate of 70% demonstrates the feasibility and acceptability of this platform in this cohort. Through the intervention, we obtained preliminary results suggestive of a high efficacy of this platform for our enrollees with low mood ratings at baseline, whose mood improved over the enrollment period, while a high mood rating was retained among those enrollees who reported high mood ratings at baseline. Second, we identified common themes of gratitude and positive mood elaborations in participant communication through qualitative text messages. These findings suggest that for those who opt to engage in communication through the platform, a text intervention for resource delivery may benefit users by improving mood and maintaining a positive mood. Further, our study reports preliminary findings that support the need for a larger randomized controlled trial of our digital mood support platform.

Prior to COVID-19, many health systems enacted clinical remote monitoring lines; however, given the current practices of quarantining and social distancing, real-time digital platforms are indispensable to patient care [9]. When designing novel well-being and mental health digital tools, it is crucial for such interventions to provide support to individuals with a low or negative mood and to those with a high or positive mood. Our study revealed an improvement in low mood ratings and the stabilization of high mood ratings among participants enrolled over 4 weeks. Although we cannot definitively conclude the cause of the observed changes in mood ratings without a comparison group, our mixed methods analysis potentially provides some valuable insights into digital patient care and mood support. Our resources, including the social engagement aspect of an interactive platform or the combination, may have led to improvements among individuals with a low mood rating and to stability for those with a high mood rating. However, our qualitative findings may provide preliminary insights into a tool for mood enhancement and well-being during the pandemic. Our intervention may have effectively supported well-being and a positive mood through “gratitude” by fostering a sense of connection and through positive reinforcement with our resources. In particular, the interactive nature of the automated text messages to the participants when asking questions, making directed statements of thanks, and offering pleasantries to the receiver may indicate that the participants found the platform interpersonal. Of note, the theme of “loneliness” was lacking, but the theme of “gratitude” was markedly apparent during communication through the platform. Lateef [10] reported that for, “EDs to make meaningful progress in enhancing patient care, safety, satisfaction, and quality, staff must listen and respond to patients and customers.” Many participants responded to the platform’s text messages as though they were responding to a peer and would ask how about our mood was and how we were doing through the pandemic. Having elicited sentiments of gratitude and connection, our intervention may serve as a digital modality for ensuring that patients and users feel heard. This pilot study shows how digital platforms and technology can bridge the gaps between individuals and community-based support.

Further large-scale randomized controlled trials are required to understand the variables involved in interpersonal communication, compared to resource delivery; nonetheless, our findings provide preliminary evidence to show that the combination of these elements may effectively enhance mood. Further, since social media exposure is reportedly associated with the exacerbation of anxiety and depression in China, which is hypothesized to be related to the “infodemic” of misinformation classified by the WHO [11], our results—suggesting the possibility that accurate, virtual resources and information can potentially benefit individuals—provide a potential means to mitigate such misinformation.

### Future Directions

To understand the effect of the COVID-19 pandemic on mental health and prepare for future public health crises, health systems and community organizations should be equipped to implement population-level efforts to address and support well-being through virtual means. The need for evidence-based mental health platforms that provide reliable information to participants was expressed even before the pandemic [12]. This study provides insights into health systems and community centers, among other stakeholders, which are faced with challenges to devise solutions to address the immediate and long-term impact of the COVID-19 pandemic on mental health issues across communities [13]. Since individual needs would likely vary through periods of social distancing, phases of lockdown, or reopening of facilities, this platform offers a promising tool to engage local community members with credible, relevant information and provide social support. Future randomized controlled trials aiming to assess and enhance the applications of this platform need to compare the present intervention group with a control group and assess the participants’ readiness to engage in support and change their mood, as this may improve subject engagement and reach.

### Limitations

This study has several limitations. First, it did not have a comparison or control group, thus limiting conclusions that can be drawn from mood improvements among participants with a low self-reported mood rating. It is possible for these participants to have improved their mood without the resources provided here, although the finding that overall mood did not change over the 7-week study period suggests otherwise. Nonetheless, these preliminary data may potentially facilitate the development of platforms focused on well-being, considering an increase in individuals experiencing mental health issues as an aftermath of the pandemic, and further highlight the need for a randomized controlled trial to assess differential mood changes between individuals who receive the intervention and those who do not. Second, this study is limited by its enrollment and retention rates. Specifically, since this study used an opt-in approach for participant enrollment, only a small subset of individuals discharged from the EDs of two hospitals were enrolled. This may have resulted in a selection bias for those who were more motivated to accept the intervention, and that mood improvement among these participants might have resulted from their readiness to change their mood rather than from the actual intervention itself. However, among the individuals who opted into this study, we observed a relatively high response rate (70%) to at least one mood rating despite limited follow-up for all four ratings. Accordingly, since recruitment essentially involved “cold” texting to most individuals discharged from the ED in a specific period, the response rate may be considered high given the pragmatic nature of the approach. Future studies are required to address the barriers to and improvements in participant retention and in engaging participants who may potentially change their mind to opt into the intervention or seek mood support. Lastly, our study used a simplified rating scale for mood, and though our results provide qualitative insights into the participants’ mood states, future studies could explore the interplay of mood ratings with depressive and anxiety symptoms. With the 70% response rate, this study highlights the feasibility of a pragmatic and completely remote mood intervention.

### Comparison With Previous Studies

The “With You” digital platform delivered real-time social or mood support, consistent with the broader goal of a digital microintervention [14]. Accordingly, our study reports on assessment parameters suggested for digital microinterventions such as user engagement, with 60% of subjects providing at least two responses to mood ratings and quantitative and qualitative data. In particular, data related to our primary outcome of interest (ie, mood) and qualitative data including the identification of users with general medical and health care concerns demonstrate the potential future implications of our example of a digital microintervention aimed toward care delivery and social engagement. Our specific results reveal a generally positive and stable mood early during the pandemic, concurrent with previous reports [15]. This explains why our findings of improved mood in our study population particularly during the pandemic are promising. Furthermore, we found that anxiety was a frequently reported negative mood elaboration in our qualitative data, concurrent with previous reports. Early during COVID-19, the Census Bureau and Centers for Disease Control and Prevention in the United States reported that one-third of Americans experienced anxiety in May 2020, the prevalence rate being almost 2-fold that reported in the 2014 census [16]. However, previous studies have reported that interventions delivered through app-based platforms effectively decreased the prevalence of depression and anxiety, with particular improvements in anxiety when the app involved interactive coaching, as is the case for apps contained in the IntelliCare platform [17]. Indeed, our study suggests the potential for digital interventions for depression and anxiety to particularly meet the needs of individuals with low mood amid the pandemic.

### Conclusions

Our findings suggest that a text messaging platform offering brief social engagement and vetted resources addressing mood support can assess and improve mood and potentially benefit end users, specifically for a likely vulnerable population—those accessing an ED during the pandemic and may otherwise not initiate their own digital engagement. This pilot study suggests that individuals with low mood, who opt to engage with the texting platform, may experience mood improvement over a 4-week period. Finally, our qualitative data may clarify the role of future interventions in supporting mood, more specifically in the context of physical health, anxiety, and gratitude. Given the current need of digital interventions and data acquisition with social distancing, these findings have implications in future measures to address community engagement and mental health support.

## Appendix

Multimedia Appendix 1 Opted in participants were texted weekly to assess mood and well-being using simple numeric scale (0-10, 0 representing worst mood and 10 as best mood) (Panel A). For participants who reported a low mood, (0-3), they received follow-up message to assess their mood compared to the previous day. If participants reported consistent low well-being scores (up to 3 days) or responded with an urgent individual need (e.g. mental health issue) they were contacted by our team and connected to a trained CHW. Mood scores four and greater received curated resources (Panel B). Panel C represents sample weekly featured resources.

Multimedia Appendix 2 Supplemental Table 1.

## Abbreviations

WHO: World Health Organization
ED: emergency department
