# Supplementary figures and images for: Improving Mood Through Community Connection and Resources Using an Interactive Digital Platform: Development and Usability Study
Source: JMIR Ment Health. 2021 Feb 26;8(2):e25834. doi: 10.2196/25834 (PMC7919843; doi:10.2196/25834)

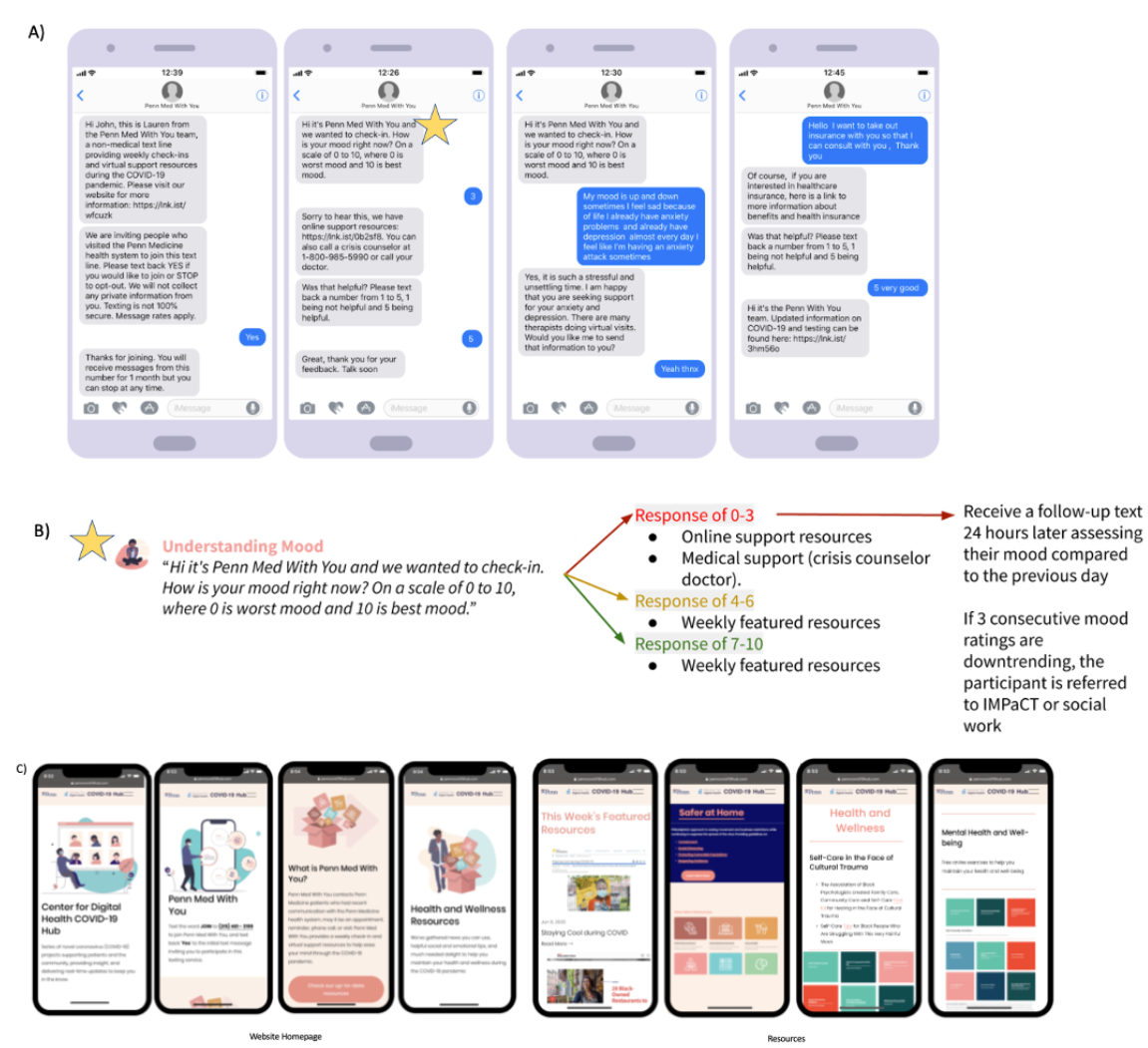

Supplement: Multimedia Appendix 1 [file mental_v8i2e25834_app1.png]

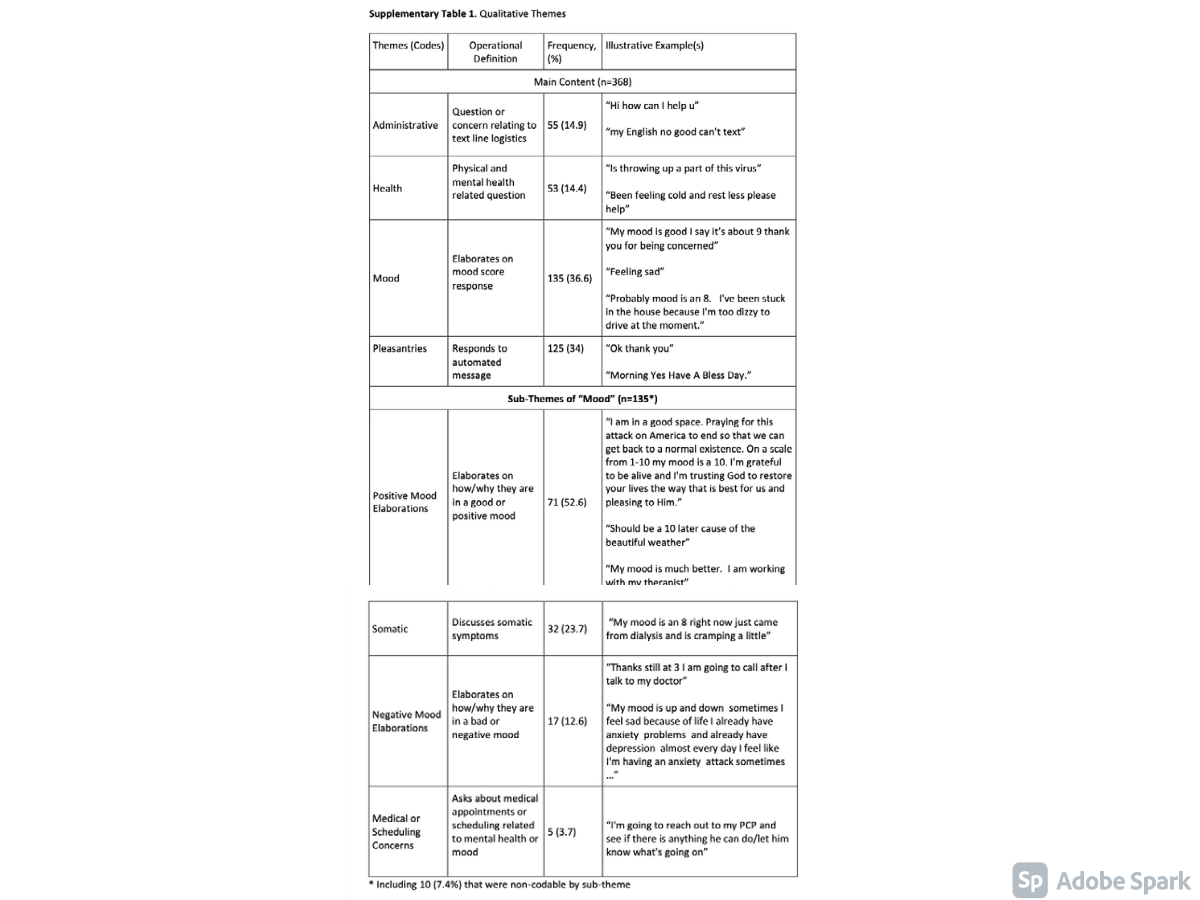

Supplement: Multimedia Appendix 2 [file mental_v8i2e25834_app2.png]
